# Application of multidisciplinary collaborative nursing intervention based on King’s Goal Attainment Theory in medical transition readiness of adolescents with chronic kidney disease

**DOI:** 10.3389/fped.2026.1936895

**Published:** 2026-08-27

**Authors:** Lili Jia, Shuo Bai, Jiwei Qi, Rong Chen, Maomao Lv, Xiaohui Xu

**Affiliations:** 1 Department of Pediatrics, Jinling Hospital, Affiliated Hospital of Medical School, Nanjing University, Nanjing, China; 2 Department of Nephrology, Jinling Hospital, Affiliated Hospital of Medical School, Nanjing University, Nanjing, China

**Keywords:** adolescents, chronic kidney disease, King's Goal Attainment Theory, medical transition, multidisciplinary collaboration, self-management

## Abstract

**Objective:**

To explore the application effect of multidisciplinary collaborative nursing intervention based on King's Goal Attainment Theory on medical transition readiness in adolescents with chronic kidney disease (CKD).

**Methods:**

A total of 88 adolescents aged 15–18 years with CKD admitted to the Department of Pediatrics of a tertiary Grade A hospital from April 2025 to September 2025 were selected. Participants were randomly divided into control group and intervention group using a random number table, with 44 cases in each group. The control group received routine pediatric nursing, while the intervention group received additional nursing intervention for medical transition readiness based on King’s Goal Attainment Theory combined with multidisciplinary team (MDT) collaboration. The intention to participate in health care, self-management ability, and medical transition readiness were compared between the two groups before intervention and 6 months after intervention.

**Results:**

After 6 months of intervention, the total score of intention to participate in health care in the intervention group (139.18 ± 20.84) was significantly higher than that in the control group (136.74 ± 21.92), with a statistically significant difference (*t* = 3.064, *P* = 0.005). The total score of self-management and medical transition readiness in the intervention group (60.41 ± 10.85) was significantly higher than that in the control group (58.25 ± 11.61), also with a statistically significant difference (*t* = 2.799, *P* = 0.01). Scores in all dimensions of the intervention group were significantly higher than those of the control group (all *P* < 0.05).

**Conclusion:**

Multidisciplinary collaborative nursing intervention based on King’s Goal Attainment Theory can effectively improve the intention to participate in health care, self-management ability, and medical transition readiness in adolescents with CKD, demonstrating clinical application value.

## Introduction

Chronic kidney disease (CKD) is a common urinary system disease in children. The prevalence of CKD in children aged 0–19 years is [15.0–126.7]/1,000,000, and more than 90% of affected children can survive into adulthood (1, 2). The period of 15–18 years of age is a critical stage for the medical transition from pediatric to adult healthcare (3). Currently, medical transition preparation for adolescents with CKD in China mainly relies on conventional health education, which is often a one-way transfer of knowledge, lacking systematic, individualized, patient-centered whole-course intervention. Furthermore, inadequate connection between pediatric and adult departments and weak multidisciplinary collaboration make it difficult to meet the core needs of physical and psychological development and independent care in adolescents (4). Insufficient transition preparation can easily lead to poor treatment adherence, increased risk of complications, and serious long-term prognosis and quality of life (5).

King’s Goal Attainment Theory (6) emphasizes perception, interaction, communication, and cooperation as its core elements, advocating that healthcare providers and patients jointly set health goals and achieve health outcomes collaboratively (7, 8). This aligns well with the core requirements of the transition period for adolescents with chronic diseases — shifting from passive care to active participation and from dependence on family to independent management. This study combines this theory with multidisciplinary collaboration to construct an intervention program for medical transition readiness and explore its application effects, as reported below.

## Methods

### Participants

During the study period, 434 adolescents with CKD were treated at our center, of whom 88 met the eligibility criteria and were enrolled in the study. Adolescents aged 15–18 years with CKD admitted to the Department of Pediatrics of a tertiary Grade A hospital from April 2025 to September 2025 were selected. Inclusion criteria: ① meeting the 2022 CKD diagnostic criteria (1) with estimated glomerular filtration rate (eGFR) stage 2–4; ② conscious with normal communication ability. Exclusion criteria: ① combined severe heart, lung, liver, or other organ failure; ② post-renal transplantation, undergoing dialysis, or in acute exacerbation stage; ③ severe mental or psychological disorders.

Sample size was calculated based on previous studies (9) with *α* = 0.05, 1−*β* = 0.90, and a 20% loss to follow-up, resulting in 44 cases per group. Participants were randomly divided into intervention group and control group (*n* = 44 each) using a random number table.

Intervention group: male 16 (36.4%), female 28 (63.6%); age 15–18 years, mean (16.37 ± 0.64) years; education: junior high school 10 (22.7%), senior high school 27 (61.4%), college in progress 7 (15.9%); eGFR stage: stage 2 in 18, stage 3 in 19, stage 4 in 7; disease duration 1–6 years, mean (3.12 ± 1.05) years; main treatment regimen: glucocorticoid alone 15, glucocorticoid+biologic agent 11, glucocorticoid + immunosuppressant 13, glucocorticoid + immunosuppressant + biologic agent 5.

Control group: male 19 (43.2%), female 25 (56.8%); age 15–18 years, mean (16.91 ± 0.47) years; education: junior high school 13 (29.5%), senior high school 20 (45.5%), college in progress 11 (25.0%); eGFR stage: stage 2 in 16, stage 3 in 21, stage 4 in 7; disease duration 1–5.8 years, mean (3.08 ± 1.12) years; main treatment regimens: glucocorticoid alone 14, glucocorticoid + biologic agent 12, glucocorticoid + immunosuppressant 12, glucocorticoid + immunosuppressant + biologic agent 6.

No significant differences in baseline characteristics were found between the two groups (all *P* > 0.05) (Table 1). All participants and their legal guardians provided written informed consent before enrollment. The study protocol was reviewed and approved by the Ethics Committee of Jinling Hospital, Affiliated Hospital of Nanjing University Medical School (Approval No. 2024DZSJJ-054). All methods were performed in accordance with the Declaration of Helsinki and relevant institutional guidelines and regulations.

**Table 1:** Baseline characteristics of participants.

Indicators	Intervention group（*n* = 44)	Control group（*n* = 44)	Statistical values	*P*
Male/Female	16/28	19/25	*χ*² = 0.562	0.453
Age	16.37 ± 0.64	16.91 ± 0.47	t = 1.892	0.062
Level of education			*χ*² = 1.215	0.545
Junior High School	10	13		
High School	27	20		
Associate degree	7	11		
eGFR staging			*χ*² = 0.217	0.897
Stage 2	18	16		
Stage 3	19	21		
Stage 4	7	7		
Duration of illness	3.12 ± 1.05	3.08 ± 1.12	t = 0.174	0.862
Treatment Plan			*χ*² = 0.186	0.98
Glucocorticoid alone	15	14		
glucocorticoid + biologic agent	11	12		
glucocorticoid + immunosuppressant	13	12		
glucocorticoid + immunosuppressant + biologic agent	5	6		

### Study methods

#### Control group

On day 1 of admission, the responsible nurse taught patients and families proper urine collection methods and provided guidance on diet and medication. From day 2, disease-related education and appropriate exercise guidance were provided. One day before discharge, the nurse assessed the patient's disease knowledge, especially medication precautions, dietary principles, and exercise amount, and instructed them to follow up regularly and respond to emergencies. Telephone follow-up was conducted 2 weeks after discharge to monitor vital signs, urine testing, urine output, medication adherence, and remind of regular follow-ups.

### Intervention group

#### MDT team formation

An 8-person MDT team was established, including 2 associate chief physicians, 2 head nurses, 1 senior nurse, 1 dietitian, 1 pharmacist, and 1 nurse. Uniform training and clear division of responsibilities were implemented. Physicians were responsible for disease assessment, treatment planning, and dynamic monitoring of disease status. Head nurses oversaw overall intervention coordination and quality control. The senior nurse performed motivational interviews, health education, and follow-up throughout the intervention. The dietitian developed and supervised individualized diet plans. The pharmacist provided medication education and adherence management, optimized regimens, and monitored adverse reactions. The nurse assisted with daily care, data collection, and follow-up records.

#### Intervention development and standardization

The intervention plan was developed through systematic literature review, two rounds of Delphi expert consultation, and a pilot study.

1. Expert consultation: 11 experts from five fields (medicine, pediatric nursing, clinical nutrition, clinical pharmacy, chronic disease nursing management) participated in two rounds of Delphi consultation. Response rates were 100% in the first round and 90.91% in the second round. Expert authority coefficient Cr = 0.83 (academic level coefficient 0.85, judgment basis coefficient 0.83, familiarity coefficient 0.81). Kendall's W = 0.272, *P* < 0.001, indicating good expert consensus. Based on expert feedback, 7 unreasonable intervention items were removed, and 12 implementation details across 6 intervention phases were refined.
2. Pilot study: Five eligible adolescent CKD patients participated in the pilot study. Implementation challenges included mismatched hospitalization days and intervention timing, rigid communication formats with adolescents, and difficulty controlling home follow-up duration. Adjustments were made to intervention timing, single-session duration, and follow-up frequency, resulting in the final 6-phase standardized medical transition preparation intervention (perception, interaction, collaboration, goal attainment, support, consolidation). Average hospitalization was 6 days.

#### Quality control indicators

Intervention quality was monitored by the head nurse using an intervention execution record form. All MDT team members received standardized training and passed assessments (training pass rate 100%). MDT quality control meetings were held every 2 weeks. Intervention consistency was evaluated, with intervention execution rate 100% and protocol adherence rate ≥95%. Follow-up response rate after discharge was 100%, and patient intervention adherence rate was ≥90%.

##### Definitions

Intervention execution rate: completed intervention items/planned items×100%.

Protocol adherence rate: fully compliant intervention items/total checked items×100%.

Patient intervention adherence: patients completing ≥3 of 4 requirements (medication check-in, home diet adherence, scheduled follow-up, peer group participation).

### Outcome measures

#### Intention to participate in health care questionnaire

The Chinese version adapted by Wu (10) was used. The questionnaire consists of 33 items across 6 dimensions: participation in medical decision-making (3 items), information interaction (9 items), treatment and care (10 items), appeal (3 items), diagnostic decision-making (5 items), and supervision (3 items). Items are scored on a 5-point Likert scale (1 = very unwilling to 5 = very willing). Total score range 33–165, with higher scores indicating better participation intention. The Cronbach's *α* coefficient of the questionnaire was 0.943.

#### Chinese version of the self-management and transition readiness questionnaire (STARx)

The STARx questionnaire, originally developed by Ferris et al. (11) for adolescents with chronic diseases, was used. The Chinese version has 18 items and 4 dimensions: medication management, health participation, patient-provider communication, and disease knowledge. The Cronbach's *α* coefficient of the total scale was 0.83. Items are scored on a 5-point Likert scale, with higher total scores indicating better transition readiness.

### Statistical analysis

SPSS 21.0 was used for data analysis. Continuous data were tested for normality (Shapiro–Wilk test, W > 0.05) and homogeneity of variance (Levene's test, F > 0.05) and presented as mean ± SD. Paired t-tests were used for within-group comparisons before and after intervention. Independent-sample t-tests were used for between-group comparisons. Categorical data were presented as numbers and percentages, and chi-square tests were used for between-group comparisons. *α* = 0.05, and *P* < 0.05 was considered statistically significant.

## Results

### Intention to participate in health care

Before intervention, no significant differences were found between the two groups in total score and dimension scores of intention to participate in health care (all *P* > 0.05). After 6 months of intervention, both groups showed significant improvement (all *P* < 0.05). The intervention group had significantly higher scores in all dimensions and total score compared to the control group (*P* < 0.05) (Table 2).

**Table 2:** Comparison of scores for intention to participate in healthcare between the two groups before and after the intervention (x ± s, points).

Dimension	Category	Before the intervention	After the intervention	Within Group *P*	Between Groups *P*
Healthcare Decisions	intervention group	13.00 ± 1.96	14.70 ± 1.03	<0.001	<0.001
	control group	12.81 ± 1.92	13.22 ± 1.81	0.019	
Information Exchange	intervention group	37.33 ± 5.34	38.44 ± 5.01	0.001	0.001
	control group	37.22 ± 5.85	37.67 ± 5.62	0.001	
Treatment and Care	intervention group	41.22 ± 7.19	42.22 ± 6.89	0.03	0.045
	control group	41.14 ± 7.86	41.71 ± 7.35	0.026	
Demands	intervention group	11.29 ± 2.85	12.07 ± 2.36	0.023	0.026
	control group	11.22 ± 2.87	11.51 ± 2.62	0.043	
Clinical Decision-Making	intervention group	20.44 ± 3.44	21.59 ± 2.77	0.01	0.01
	control group	20.29 ± 3.29	20.85 ± 3.03	0.006	
Interpellation and Oversight	intervention group	12.51 ± 2.37	13.55 ± 1.78	0.012	0.011
	control group	12.37 ± 2.23	12.96 ± 1.87	<0.001	
Total score	intervention group	135.92 ± 22.17	139.18 ± 20.84	0.003	0.005
	control group	136.07 ± 21.95	136.74 ± 21.92	0.026	

### Self-management and transition readiness

Before intervention, no significant differences were found between the two groups in STARx total score and dimension scores (all *P* > 0.05). After 6 months, both groups showed significant improvement (all *P* < 0.05). The intervention group had significantly higher scores in all dimensions and total score compared to the control group (*P* < 0.05) (Table 3).

**Table 3:** Comparison of self-management and transition to primary care scores between the two groups before and after intervention (x ± s, points).

Dimension	Category	Before the intervention	After the intervention	Within Group *P*	Between Groups *P*
Drug Management	intervention group	21.74 ± 3.47	23.25 ± 2.19	0.002	0.004
	control group	21.59 ± 3.28	22.44 ± 2.77	0.001	
Health Engagement	intervention group	15.62 ± 5.06	17.11 ± 4.77	0.007	0.007
	control group	15.55 ± 4.95	16.11 ± 4.84	0.005	
Doctor-Patient Communication	intervention group	12.77 ± 3.77	14.29 ± 2.94	0.003	0.005
	control group	12.88 ± 3.69	13.25 ± 3.41	0.022	
Disease Information	intervention group	7.07 ± 2.35	8.18 ± 1.64	0.003	0.004
	control group	7.06 ± 2.25	7.59 ± 1.82	0.004	
Total score	intervention group	57.22 ± 12.37	60.41 ± 10.85	0.011	0.01
	control group	56.96 ± 12.33	58.25 ± 11.61	0.005	

## Discussion

### Multidisciplinary nursing intervention based on King’s Goal Attainment Theory improves transition readiness in adolescents with CKD

After 6 months, the intervention group had significantly higher STARx total and dimension scores than the control group (*P* < 0.05), indicating that the MDT intervention based on King's theory systematically improves medical transition readiness in adolescents with CKD. This trend is consistent with the findings of An Liqiao et al. (12), confirming the effectiveness of MDT models in improving transition readiness in adolescents with chronic diseases. Compared to conventional MDT interventions focusing primarily on disease management and health education, our intervention simultaneously targeted intrinsic participation motivation and external professional management, better aligning with adolescent psychological development and enhancing patient initiative.

Adolescents with CKD often have insufficient disease knowledge, poor self-management, and anxiety about adult healthcare settings. Conventional one-way education is inadequate (13). This study overcomes the limitations of previous interventions that focused only on knowledge and skills (14, 15). Using King’s theory (perception, interaction, collaboration, goal attainment) as the framework, we integrated psychological support, family guidance, and cross-departmental connections. The sequential mechanism of psychological arousal, self-efficacy enhancement, independent decision-making, and behavior consolidation shifted care from passive education to collaborative goal attainment. MDT collaboration integrated disease management, medication guidance, and nutritional support, compensating for shortcomings of traditional nursing models.

The perception phase accurately identified individual needs and transition barriers, awakening autonomous willingness. The interaction phase built trust and enhanced disease mastery through two-way communication. The collaboration phase strengthened patient agency through shared goal setting. The goal attainment phase translated motivation into stable behavior through practical training. The support and consolidation phases provided a full-cycle closed-loop system. This approach aligns with adolescent identity development, effectively reducing family overprotection, stigma, and environmental fear, upgrading transition readiness from knowledge memorization to internalized competence. Future clinical practice should develop stratified assessment tools based on STARx, standardize training modules such as scenario simulation and behavior check-ins, and promote routine pediatric-adult joint clinics.

### The intervention improves intention to participate in health care in adolescents with CKD

After 6 months, the intervention group had significantly higher total and dimension scores for intention to participate in health care than the control group (*P* < 0.05). This finding aligns with Yu Genzhen et al. (16), confirming that individualized assessment and interactive interventions effectively improve participation intention. Adolescents with CKD are in a critical stage of identity vs. role confusion, and disease stigma, transition anxiety, and family over-dependence together inhibit participation intention (17), which conventional one-way nursing fails to address.

With King’s theory as the core, our intervention established a closed-loop system of cognitive arousal, emotional support, autonomous participation, and behavioral consolidation. Multidisciplinary assessment identified participation barriers, two-way communication reduced anxiety and increased confidence, shared decision-making strengthened patient agency, and scenario simulation and positive reinforcement translated intention into stable behavior. Family role adjustment, encouraging parents to appropriately let go, provided crucial social support for adolescent autonomy, enabling the shift from “passive compliance” to “active engagement.” MDT collaboration supported the intervention by providing consistent, multi-disciplinary information on disease, medication, and nutrition, enhancing understanding and trust. Shared decision-making models and visual decision aids should be promoted in clinical practice.

### Practical significance in China

Transition preparation nursing for adolescents with CKD in China is still in early stages, often limited to discharge guidance and short-term follow-up, resulting in fragmented interventions, inadequate cross-departmental connections, and lack of individualized whole-course management. Our theory-guided, MDT-based, 6-phase closed-loop intervention is more systematic, continuous, and professional than traditional models, offering a replicable, quality-controllable, and generalizable practice template for pediatric nephrology departments in tertiary hospitals, with important implications for standardizing transition preparation for adolescents with chronic diseases in China.

## Conclusion

Multidisciplinary collaborative nursing intervention based on King’s Goal Attainment Theory significantly improves intention to participate in health care, self-management ability, and medical transition readiness in adolescents with chronic kidney disease, providing a scientific, standardized, and feasible practice plan for transition preparation in this population.

## Limitations and future directions

This study is a single-center RCT with a limited sample size. Outcome measures rely mainly on subjective scales, lacking objective indicators such as renal function and actual transition success rates. Moreover, the MDT model may be difficult to generalize in primary care settings due to human resource and cross-departmental collaboration constraints. Future studies should conduct multicenter, large-sample, long-term follow-up research with objective outcome measures. A simplified, stratified version of the intervention should be developed for primary care settings to further improve the transition preparation nursing system for adolescents with CKD in China.

## Data Availability

The original contributions presented in the study are included in the article/Supplementary Material, further inquiries can be directed to the corresponding author.
